# Distinct Disruptive Patterns of Default Mode Subnetwork Connectivity Across the Spectrum of Preclinical Alzheimer’s Disease

**DOI:** 10.3389/fnagi.2019.00307

**Published:** 2019-11-13

**Authors:** Chen Xue, Baoyu Yuan, Yingying Yue, Jiani Xu, Siyu Wang, Meilin Wu, Nanxi Ji, Xingzhi Zhou, Yilin Zhao, Jiang Rao, Wenjie Yang, Chaoyong Xiao, Jiu Chen

**Affiliations:** ^1^Department of Radiology, The Affiliated Brain Hospital of Nanjing Medical University, Nanjing, China; ^2^Institute of Neuropsychiatry, The Affiliated Brain Hospital of Nanjing Medical University, Fourth Clinical College of Nanjing Medical University, Nanjing, China; ^3^Department of Neurology, Affiliated ZhongDa Hospital, School of Medicine, Southeast University, Nanjing, China; ^4^Department of Psychosomatics and Psychiatry, ZhongDa Hospital, School of Medicine, Southeast University, Nanjing, China; ^5^Department of Rehabilitation, The Affiliated Brain Hospital of Nanjing Medical University, Nanjing, China; ^6^Institute of Brain Functional Imaging, Nanjing Medical University, Nanjing, China

**Keywords:** amnestic mild cognitive impairment, non-amnestic mild cognitive impairment, subjective cognitive decline, default mode network, functional connectivity

## Abstract

**Background**: The early progression continuum of Alzheimer’s disease (AD) has been considered to advance through subjective cognitive decline (SCD), non-amnestic mild cognitive impairment (naMCI), and amnestic mild cognitive impairment (aMCI). Altered functional connectivity (FC) in the default mode network (DMN) is regarded as a hallmark of AD. Furthermore, the DMN can be divided into two subnetworks, the anterior and posterior subnetworks. However, little is known about distinct disruptive patterns in the subsystems of the DMN across the preclinical AD spectrum. This study investigated the connectivity patterns of anterior DMN (aDMN) and posterior DMN (pDMN) across the preclinical AD spectrum.

**Methods**: Resting-state functional magnetic resonance imaging (rs-fMRI) was used to investigate the FC in the DMN subnetworks in 20 healthy controls (HC), eight SCD, 11 naMCI, and 28 aMCI patients. Moreover, a correlation analysis was used to examine associations between the altered connectivity of the DMN subnetworks and the neurocognitive performance.

**Results**: Compared to the HC, SCD patients showed increased FC in the bilateral superior frontal gyrus (SFG), naMCI patients showed increased FC in the left inferior parietal lobule (IPL), and aMCI patients showed increased FC in the bilateral IPL in the aDMN; while SCD patients showed decreased FC in the precuneus, naMCI patients showed increased FC in the left middle temporal gyrus (MTG), and aMCI patients also showed increased FC in the right middle frontal gyrus (MFG) in the pDMN. Notably, the FC between the ventromedial prefrontal cortex (vmPFC) and the left MFG and the IPL in the aDMN was associated with episodic memory in the SCD and aMCI groups. Interestingly, the FC between the posterior cingulated cortex (PCC) and several regions in the pDMN was associated with other cognitive functions in the SCD and naMCI groups.

**Conclusions**: This study demonstrates that the three preclinical stages of AD exhibit distinct FC alternations in the DMN subnetworks. Furthermore, the patient group data showed that the altered FC involves cognitive function. These findings can provide novel insights for tailored clinical intervention across the preclinical AD spectrum.

## Introduction

Mild cognitive impairment (MCI), which is divided into amnestic mild cognitive impairment (aMCI) and non-amnestic mild cognitive impairment (naMCI; Grundman et al., 2004; Kim et al., 2015; Makovac et al., 2018), is regarded as the intermediate stage between healthy aging and dementia. Neuroimaging studies have demonstrated that aMCI, characterized by memory decline, has a high probability of developing into Alzheimer’s disease (AD) dementia (Rossetto et al., 2018; Chen et al., 2019a). Moreover, several previous studies have indicated that naMCI might be an intermediate stage between health and aMCI/AD (Lee et al., 2018; Oltra-Cucarella et al., 2018). Furthermore, subjective cognitive decline (SCD), as an earlier stage of MCI, refers to the elderly with a normal cognitive performance level and no objective signs of cognitive impairment who subjectively think they are cognitively impaired (Funaki et al., 2019; Hu et al., 2019). Thus, converging evidence suggests that the development of AD may partly progress through a continuum from SCD to MCI and eventually to AD (Berger-Sieczkowski et al., 2019). This could mean that SCD, naMCI, and aMCI can be considered as a spectrum of preclinical AD, which may have a different topography of pathological involvement during different disease stages. Therefore, it is of great significance to promote our understanding of abnormal patterns across the preclinical AD spectrum, and it is particularly important to provide a tailored clinical intervention across the preclinical AD spectrum.

In recent years, resting state functional magnetic resonance imaging (rs-fMRI) has become the main means of cognitive research, while the default mode network (DMN) has been the most studied network (Cai et al., 2017; Banks et al., 2018). The DMN, anatomically distributed in different areas of the brain, can be divided into two subnetworks, the anterior and posterior subnetworks. The anterior subnetwork (the anterior DMN, aDMN) is mainly composed of the ventromedial prefrontal cortex (vmPFC), which is involved in self-referential mental idealization, and the posterior subnetwork (the posterior DMN, pDMN), which consists of the posterior cingulated cortex (PCC) and is involved in episodic memory retrieval (Yang et al., 2017; Wang et al., 2018). Some studies have shown that amyloid deposition is most likely to occur in the medial prefrontal cortex and PCC, which belong to the DMN (Wang et al., 2013). Further results have indicated that regions belonging to the DMN were affected early in the process of developing to AD, and functional connectivity (FC) changes in the DMN have been reported as predictors of AD conversion (Crockett et al., 2017; Scherr et al., 2019). In addition, the altered FC of the DMN is related to the change of cognitive performance (Joshi et al., 2018). Notably, the study of altered FC in DMN subnetworks might provide a pattern to explain the pathophysiology of AD.

Several neuroimaging studies have indicated increased FC in the DMN in SCD patients compared to healthy controls (HC), especially between pDMN and the medial temporal memory system (MTMS; Verfaillie et al., 2018). In naMCI, there tends to be a change in connectivity between the hippocampus and the PCC, and the PCC is an important area in the DMN (Dunn et al., 2014; Prieto Del Val et al., 2016). Furthermore, there is no statistical difference in the DMN intra-connectivity between naMCI and aMCI (Dunn et al., 2014). To our knowledge, there have been no studies of specific default mode subnetworks in naMCI. Additionally, in aMCI, some investigations have found increased FC in the aDMN and decreased FC in the pDMN (Wu et al., 2016); the increased FC in the aDMN was considered to be a compensatory addition of cognitive function to sustain task performance (Qi et al., 2010; Damoiseaux et al., 2012; Dunn et al., 2014). Taken together, these observations suggest that the patterns of impairment in the anterior and posterior subnetworks in these patients seem to differ. However, prior studies have focused on the specific diseases, and very little is known about whether there is a progression of the DMN subnetwork impairment pattern across the preclinical AD spectrum or potentially a corresponding progression of cognitive impairment.

Therefore, the objective of the current study is to analyze changes in the FC patterns of the DMN subnetworks across the preclinical AD spectrum, including SCD, naMCI, and aMCI, and to further investigate the relationship between the disruptive patterns of the DMN subnetworks and cognitive function. We hypothesized that there exists a distinct alteration of the DMN subnetworks in the three preclinical stages of AD and that the altered patterns in the aDMN and pDMN may contribute to different levels of cognitive impairment across the preclinical AD spectrum (Yuan et al., 2016b).

## Materials and Methods

### Subjects

The present study recruited 79 elderly individuals: 21 HC, 10 SCD, 15 naMCI, and 33 aMCI individuals were selected to participate in our research from hospitals, communities, and a broadcasting station. However, 12 of the participants were excluded due to no MRI data (*n* = 10) and excessive head motion (cumulative translation or rotation >3.0 mm or 3.0°, *n* = 2). As a result, the study included 67 subjects in total (20 HC, 8 SCD, 11 naMCI, and 28 aMCI). The study participants had to meet the following criteria: (1) 40–80 years old; (2) secondary school education or higher; (3) right-handedness; (4) Han Chinese language speakers; (5) no history of serious diseases that could influence cerebral function, such as severe brain injury, brain tumor, brain hemorrhage, brain infarction, white matter disease, neurologic, psychiatric, and systemic illnesses; and (6) no history of psychoactive medications (Dillen et al., 2017; Vecchio et al., 2018).

The inclusion criteria for HC were: (1) no memory complaints; (2) normal cognitive performance of age- and education-matched volunteers; and (3) Clinical Dementia Rating (CDR) = 0 (Chen et al., 2016b; Gu et al., 2018; Yan et al., 2018).

The inclusion criteria for SCD were based on the published SCD criteria proposed by the Subjective Cognitive Decline Initiative (SCD-I): (1) always complained of memory problems; (2) Subjective Cognitive Decline Questionnaire (SCD-Q) > 5; (3) normal cognitive performance of age- and education-matched norms; and (4) CDR = 0 (Dillen et al., 2017; Yan et al., 2018; Cedres et al., 2019).

The inclusion criteria for naMCI were: (1) normal overall cognitive function as evidenced by: CDR = 0.5, Mini-Mental State Examination (MMSE) score ≧ 26, the Montreal Cognitive Assessment (MoCA) ≧ 26, Mattis Dementia Rating Scale-2 (MDRS-2) ≧ 120, and Hamilton Depression Rating Scale (HAMD) ≦ 7; and (2) objective impairment in at least one cognitive domain except memory function, including visual spatial function, executive function, and information processing speed (Dunn et al., 2014).

The inclusion criteria for aMCI were: (1) patients complained of memory impairment of at least 3 months or relatives confirmed that the memory impairment had lasted for more than 3 months; (2) objective memory performance documented by an Auditory Verbal Memory Test-delayed recall (AVLT-DR) score within ≦ 1.5 standard deviation (SD) of same age- and education-adjusted norms; (3) normal overall cognitive function as described for naMCI; and (4) not demented (Dunn et al., 2014; Chen et al., 2019b; Huang et al., 2019; Zhang et al., 2019).

The study was approved by the responsible Human Participants Ethics Committee of the Affiliated Brain Hospital of Nanjing Medical University. Written informed consent was obtained from all participants.

### Neurocognitive Assessments

All participants underwent comprehensive and standard neurocognitive assessments to evaluate their cognitive function, including general cognitive functions, episodic memory, executive function, information processing speed, and visual spatial domains (Gu et al., 2017; Gao et al., 2018). These assessments include the MMSE, the ADL, the MDRS-2, the MoCA, the SCD-Q, the CDR, the Hachinski Ischemic Scale (HIS), the HAMD, the Auditory Verbal Learning Test (AVLT; including the AVLT-immediate, the AVLT-5 min delay, and the AVLT-20 min delay), the Rey Complex Figure Test (CFT) delay, the Logical Memory Test (LMT), the CFT, the Clock-Drawing Test (CDT), the Boston Naming Test, the Category Verbal Fluency Test (including the CVFT-animals and the CVFT-objects), the Symbol Digit Modalities Test, the part A and B of the Trail Making Test (TMT), the Digit Span Test (including the DS forward and the DS backward), part A, B, and C of the Stroop Test, and the Semantic Similarity Test. These scales are widely used in cognitive assessment, verified by two senior neuropsychologists and evaluated by experienced clinicians.

### MRI Data Acquisition

All magnetic resonance imaging (MRI) data were acquired using a 3.0 Tesla Verio Siemens scanner with an 8-channel head-coil in the Affiliated Brain Hospital of Nanjing Medical University. Resting-state functional images were collected when participants were instructed to rest with their eyes open, to not fall asleep, and to not think of anything in particular. The gradient-echo echo-planar imaging (GRE-EPI) sequence included 240 volumes (Chen et al., 2016a). The parameters were: repetition time (TR) = 2,000 ms, echo time (TE) = 30 ms, number of slices = 36, thickness = 4.0 mm, gap = 0 mm, matrix = 64 × 64, flip angle (FA) = 90°, field of view (FOV) = 220 mm × 220 mm, acquisition bandwidth = 100 kHz, and voxel size = 3.4 × 3.4 × 4 mm^3^. The imaging took approximately 8 min.

High-resolution T1-weighted images were acquired by 3D magnetization-prepared rapid gradient-echo (MPRAGE) sequence (Chen et al., 2016a). The parameters were: TR = 1,900 ms, TE = 2.48 ms, inversion time (TI) = 900 ms, number of slices = 176, thickness = 1.0 mm, gap = 0.5 mm, matrix = 256 × 256, FA = 9°, FOV = 256 mm × 256 mm, and voxel size = 1 × 1 × 1 mm^3^. The imaging took approximately 4.26 min.

### Image Preprocessing

All fMRI data were preprocessed by MATLAB2013b^1^ and Data Processing and Analysis for Brain Imaging (DPABI), which is based on Statistical Parametric Mapping (SPM8)^2^. The first 10 volumes were discarded to reduce the instability of the MRI signal. Corrections were performed for the intra-volume acquisition time differences among slices and inter-volume motion effects during the scan. Participants with excessive head motion (cumulative translation or rotation >3.0 mm or 3.0°) were excluded. Then, we chose affine regularization in European segmentation and nuisance covariate regression with 24 motion parameters, a global signal, a white matter signal, and a cerebrospinal fluid signal (Fox et al., 2009). Data were filtered at 0.01–0.08 Hz to reduce the effect of the low-frequency drift and high-frequency physiological noise, such as breathing and heartbeats (Chen et al., 2016a). Next, to spatially normalize the fMRI data, we used T1 image unified segmentation and resampled to an isotropic voxel size of 3 mm. At last, spatial smoothing using a 6-mm full-width half-maximum Gaussian kernel (Cha et al., 2013) and detrending were applied to reduce spatial noise and differences in anatomical structures among subjects. Stringent quality assurance measures were performed as described in previous studies to reduce the impact of head motion on the rsfMRI results (Power et al., 2012; Van Dijk et al., 2012).

### Functional Connectivity Analysis

A seed-based FC analysis was performed to explore the alternation of DMN subnetworks. To identify the seed region in the present study, two 10-mm spherical regions of interest centered in the vmPFC (MNI space: 0, 52, −6, Brodmann area 10) and PCC (MNI space: 0, −53, 26, Brodmann area 31) were created (Zhang and Raichle, 2010; Yuan et al., 2016b). Individual mean time series were extracted based on the coregistered seed region as the reference time series, and then a voxel-wise cross-correlation analysis was carried out between the seed region and the whole brain within the gray matter (GM) mask. We used a Fisher’s r-to-z transformation to improve the normality of the correlation coefficients. Then, we obtained the vmPFC subnetwork (aDMN) and the PCC subnetwork (pDMN).

### Statistical Analyses

The Statistical Package for the Social Sciences (SPSS) software version 22.0 (IBM, Armonk, New York, NY, USA) was used for statistical analyses. The analysis of variance (ANOVA), the multimodal general linear model (GLM), and the chi-square test were conducted to compare the demographic and neurocognitive data among groups, including the HC, SCD, naMCI, and aMCI. The Bonferroni correction was used for *post hoc* comparisons. The *P*-value was set as <0.05 for significant differences.

A one-way ANOVA analysis was performed to compare the differences in FC in both the aDMN and pDMN among four groups after controlling for the effects of age, gender, level of education, and GM volumes. As suggested in a previous study, the non-parametric permutation test can precisely control the false positive rate in the cluster-level inference; therefore, we set the permutation times at 1,000 (Smith and Nichols, 2009). The corrected *p*-value < 0.05 was set for statistical significance and the cluster size >200 voxels (5,400 mm^3^) was applied for multiple comparisons at the voxel level. The two-sample *T*-test was used for *post hoc* comparisons with the mask resulted from ANOVA analyses after controlling for the effects of age, gender, level of education, and GM volumes. We set the significance level with a family-wise error (TFCE-FWE) corrected cluster *p* < 0.05 and the cluster size >10 voxels (270 mm^3^). The FCs of significantly altered regions were extracted with a Resting-State fMRI Data Analysis Toolkit (REST)^3^ and were later used for correlation analyses. The correlation analyses were conducted to reveal the relationships between the altered FCs of the DMN and cognitive domains after controlling for the effects of age, gender, and level of education (*p* < 0.05, Bonferroni-corrected).

It is worth mentioning that, we grouped the neuropsychological tests into four cognitive domains (Gu et al., 2017; Gao et al., 2018). Episodic memory data were mainly derived from AVLT-20-min DR, LMT-20-min DR, and CFT-20-min DR. The information processing speed dates were mainly acquired from DSST, TMT-A, Stroop A, and Stroop B. Visuospatial function data were mainly extracted from the CFT and CDT. Executive function data were mainly obtained from VFT, DST-backward, TMT-B, Stroop C, and Semantic Similarity. The individual raw score of each neuropsychological test was transformed to normalized *Z* scores. Subsequently, the normalized *Z* score was averaged to calculate the composite *Z* score of each cognitive domain (Xie et al., 2012; Chen et al., 2016b; Yuan et al., 2016b).

## Results

### Demographic and Neurocognitive Characteristics

The demographic and neurocognitive information of all participants, including 21 HC (mean age 57.52 ± 8.072), 10 SCD (mean age 63.10 ± 8.774), 15 naMCI (mean age 63.87 ± 8.568), and 33 aMCI (mean age 66.03 ± 8.579) individuals, can be found in Table 1. As expected, our results showed significant differences in cognitive performance. Compared to HC, the aMCI group exhibited significantly lower MMSE, MDRS-2, MoCA scores, episodic memory, information processing speed, executive function, and visuospatial function, though it exhibited significantly higher SCD-Q scores; the naMCI group exhibited significantly lower information processing speed and executive function. Compared to SCD group, the aMCI group exhibited significantly lower episodic memory and executive function; the naMCI group exhibited significantly lower information processing speed, executive function, and education level. Compared to naMCI group, the aMCI group exhibited significantly lower episodic memory scores. Last, the SCD group had the highest SCD-Q scores (all *p* < 0.05).

**Table 1:** Demographics and clinical measures of HC and patients with SCD, naMCI, and aMCI.

	HC	SCD	naMCI	aMCI	*F*-values (*χ*^2^)	*p*-values
	*n* = 21	*n* = 10	*n* = 15	*n* = 33		
Age (years)	57.52 (8.072)	63.10 (8.774)	63.87 (8.568)	66.03 (8.579)*	4.388	0.007
Gender (male/female)	7/14	4/10	6/9	11/22	6.696	0.01
Education level (years)	12.05 (2.747)	13.85 (1.827)	10.60 (2.694)**	11.06 (3.358)	3.086	0.032
FD	0.0856 (0.04929)	0.0663 (0.04287)	0.1181 (0.05258)	0.0908 (0.07461)	1.074	0.367
MMSE scores	28.81 (1.209)	27.70 (1.160)	28.27 (1.710)	26.88 (2.027)*	6.156	0.001
MDRS-2	141.00 (2.915)	138.80 (3.393)	137.07 (3.432)	134.82 (7.970)*	5.193	0.003
MoCA	26.20 (2.624)	25.00 (3.432)	24.14 (2.931)	22.40 (3.379)*	5.357	0.002
SCD-Q	3.07 (1.591)	6.05 (0.725)*	4.36 (2.161)	5.45 (1.690)*	9.033	<0.001
Composite *Z* scores of each cognitive domain					
Episodic memory	0.4953 (0.58422)	0.5788 (0.48495)	0.3315 (0.53204)	−0.6245 (0.54907)*/**/****	25.373	<0.001
Information processing speed	0.4525 (0.75906)	0.4152 (0.46834)	−0.4064 (0.65820)*/**	−0.2252 (0.77552)*	6.535	0.001
Executive function	0.4511 (0.72800)	0.4369 (0.50400)	−0.2714 (0.48974)*/**	−0.2493 (0.61376)*/**	7.974	<0.001
Visuospatial function	0.2642 (0.56747)	0.4183 (0.44671)	−0.1231 (0.68174)	−0.2438 (1.01123)	2.79	0.046

Numbers are given as means (standard deviation, SD) unless stated otherwise. Scores reflect the number of correct items unless stated otherwise. Significant group differences were found at *p* < 0.05 (ANOVA test and Multivariable general linear model), Bonferroni corrected. FD, framewise displacement; MMSE, Mini-Mental State Exam; MDRS-2, Mattis Dementia Rating Scale-2; MoCA, the Montreal Cognitive Assessment test; SCD-Q, Subjective Cognitive Decline Questionnaire. *Compared to HC, **compared to SCD, ****compared to naMCI; HC, healthy controls; SCD, subjective cognitive decline; aMCI, amnestic mild cognitive impairment; naMCI, non-amnestic mild cognitive impairment.

### Altered FC Patterns of DMN Subnetworks in Patients with SCD, naMCI, and aMCI

**Figure 1:**
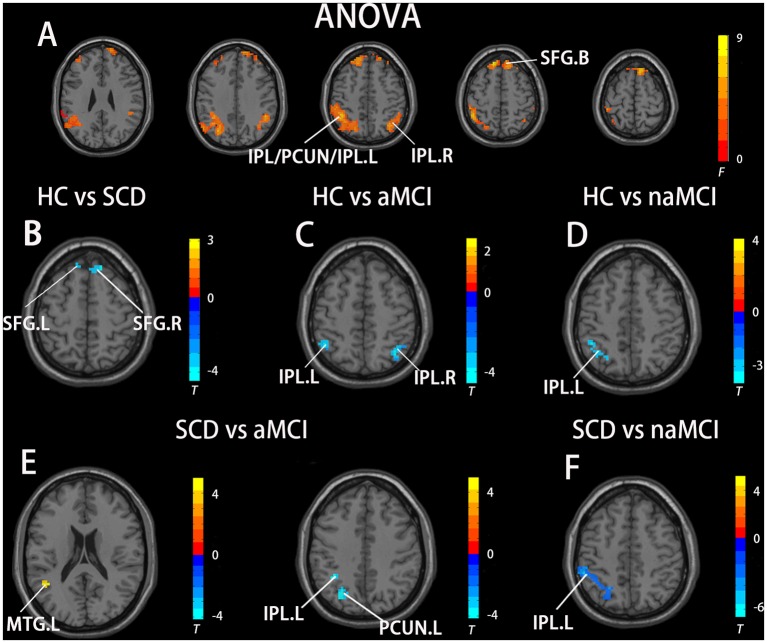
Brain regions exhibiting significant differences in functional connectivity (FC) of the anterior default mode network (DMN) subnetwork [the ventromedial prefrontal cortex (vmPFC) subnetwork] based on analysis of variance (ANOVA) analysis and two-sample *T*-tests. Age, gender, and years of education were used as covariates for all these results. **(A)** Brain regions showing significant differences in FC of the anterior DMN subnetwork between HC, patients with SCD, patients with naMCI, and patients with Alzheimer’s disease (AD; *p* < 0.05, the cluster size > 200 voxels). **(B–F)** The results of *post hoc* two-sample *T*-tests in voxel-wise analysis (TFCE-FWE corrected, cluster size ≥ 10, *p* < 0.05). aMCI, amnestic mild cognitive impairment; naMCI, non-amnestic mild cognitive impairment; SCD, subjective cognitive decline; HC, healthy controls; IPL, inferior parietal lobule; PCUN, precuneus; SFG, superior frontal gyrus; MTG, middle temporal gyrus; L, left; R, right.

In the aDMN subnetwork, the ANOVA analysis showed five significantly altered brain regions among the groups, including the bilateral inferior parietal lobule (IPL), left precuneus (PCUN), left middle temporal gyrus (MTG), and bilateral superior frontal gyrus (SFG). Compared to the HC, the SCD patients showed significantly increased FC in the bilateral SFG, the naMCI individuals showed increased FC in the left IPL, and the aMCI patients showed increased FC in the bilateral IPL. Compared to the SCD group, the aMCI group showed increased FC in the left IPL, left MTG, and left PCUN, while the naMCI group showed increased FC in the left IPL. It is worth noting that, compared to the HC, the SCD, naMCI, and aMCI groups all showed increased FC in the aDMN (TFCE-FWE corrected, cluster size ≥ 10, *p* < 0.05). All results are after controlling the effects of age, gender, level of education, and GM volumes (see Figure 1 and Table 2).

**Table 2:** The differences in functional connectivity in the ventromedial prefrontal cortex (vmPFC) subnetwork.

Region(aal)	Peak MNI coordinate			F/t	Cluster number
	*x*	*y*	*z*		
**ANOVA**					
L Inferior Parietal Lobule/Precuneus/Middle Temporal Gyrus	−45	−51	51	8.7072	1,178
R Inferior Parietal Lobule	48	−45	36	7.3382	263
B Superior Frontal Gyrus	12	24	66	8.1821	498
**SCD>HC**					
L Superior Frontal Gyrus	−12	36	51	4.4635	20
R Superior Frontal Gyrus	12	33	54	4.1155	33
**aMCI>HC**					
R Inferior Parietal Lobule	36	−66	45	4.5565	122
L Inferior Parietal Lobule	−45	−51	51	4.0654	23
**naMCI>HC**					
L Inferior Parietal Lobule	−42	−45	42	3.931	82
**aMCI>SCD**					
L Middle Temporal Gyrus	−45	−51	21	4.9974	15
L Precuneus	−27	−60	33	4.1604	62
L Inferior Parietal Lobule
**naMCI>SCD**	−36	−45	42	4.0831	68
L Inferior Parietal Lobule	−45	−45	57	6.6488	262

*The x, y, z coordinates are the primary peak locations in the MNI space. Cluster size > 200 voxels in ANOVA analysis, *p* < 0.05; Cluster size > 10 voxels in two-sample T-test, *p* < 0.05, TFCE-FWE corrected*.

**Figure 2:**
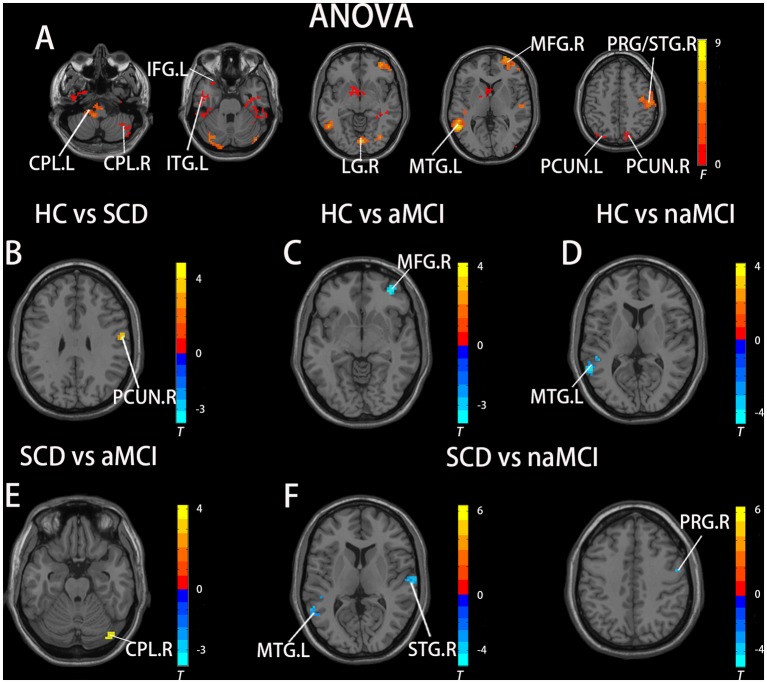
Brain regions exhibiting significant differences in FC of the posterior DMN subnetwork [the posterior cingulated cortex (PCC) subnetwork] based on ANOVA analysis and two-sample *T*-tests. Age, gender, and years of education were used as covariates for all these results. **(A)** Brain regions showing significant differences in FC of the posterior DMN subnetwork between HC, patients with SCD, patients with naMCI, and patients with AD (*p* < 0.05, the cluster size > 200 voxels). **(B–F)** The results of *post hoc* two-sample *T*-tests in voxel-wise analysis (TFCE-FWE corrected, cluster size ≥ 10, *p* < 0.05). aMCI, amnestic mild cognitive impairment; naMCI, non-amnestic mild cognitive impairment; SCD, subjective cognitive decline; HC, healthy controls; CPL, cerebellum posterior lobe; ITG, inferior temporal gyrus; LG, lingual gyrus; IFG, inferior frontal gyrus; MTG, middle temporal gyrus; MFG, middle frontal gyrus; PRG, precentral gyrus; STG, superior temporal gyrus; PCUN, precuneus; L, left; R, right.

In the pDMN subnetwork, the ANOVA analysis showed 11 significantly altered brain regions among the groups, including the bilateral cerebellum posterior lobe (CPL), right inferior temporal gyrus (ITG), right lingual gyrus (LG), left inferior frontal gyrus (IFG), left MTG, right middle frontal gyrus (MFG), right precentral gyrus (PRG), right superior temporal gyrus (STG), and bilateral PCUN. Compared to the HC, the SCD patients showed decreased FC in the right PCUN, the naMCI individuals showed increased FC in the left MTG, and the aMCI patients showed increased FC in the left MFG. Compared to the SCD group, the naMCI group showed increased FC in the left MTG, right STG, and right PCG, while the aMCI group showed decreased FC in the right CPL. Notably, compared to the HC, the SCD group showed decreased FC, while both the naMCI and aMCI groups showed increased FC in the pDMN (TFCE-FWE corrected, cluster size ≥ 10, *p* < 0.05). All results are after controlling the effects of age, gender, level of education, and GM volumes (see Figure 2 and Table 3).

**Table 3:** The differences in functional connectivity in the posterior cingulated cortex (PCC) subnetwork.

Region(aal)	Peak MNI coordinate			F/t	Cluster number
	*x*	*y*	*z*		
**ANOVA**					
L Cerebellum Posterior Lobe	−12	−39	−51	6.7851	211
R Cerebellum Posterior Lobe	51	−60	−51	0.22507	425
L Inferior Temporal Gyrus	−48	−3	−48	0.23658	212
R Lingual Gyrus	−3	−81	−6	7.5879	314
L Inferior Frontal Gyrus	−27	18	−27	0.21662	281
L Middle Temporal Gyrus	−57	−51	3	9.2035	380
R Middle Frontal Gyrus	30	66	6	6.9772	248
R Precentral Gyrus/Superior Temporal Gyrus	54	−18	48	5.364	404
R Precuneus	−15	−81	54	0.21035	290
L Precuneus	−15	−93	36	0.18706	210
**HC>SCD**					
R Precuneus	57	−12	30	4.9182	19
**aMCI>HC**					
R Middle Frontal Gyrus	36	48	−6	3.8891	69
**naMCI>HC**					
L Middle Temporal Gyrus	−60	−51	9	4.5979	87
**SCD>aMCI**					
R Cerebellum Posterior Lobe	42	−78	−21	4.2026	24
**naMCI>SCD**					
L Middle Temporal Gyrus	−51	−48	−3	5.0983	130
R Superior Temporal Gyrus	57	−15	3	4.6523	50
R Precentral Gyrus	54	0	45	4.434	19

*The x, y, z coordinates are the primary peak locations in the MNI space. Cluster size > 200 voxels in ANOVA analysis, *p* < 0.05; Cluster size > 10 voxels in two-sample *T*-test, *p* < 0.05, TFCE-FWE corrected*.

### Behavioral Significance of the Disrupted Functional Connectivity of DMN Subnetworks

**Figure 3:**
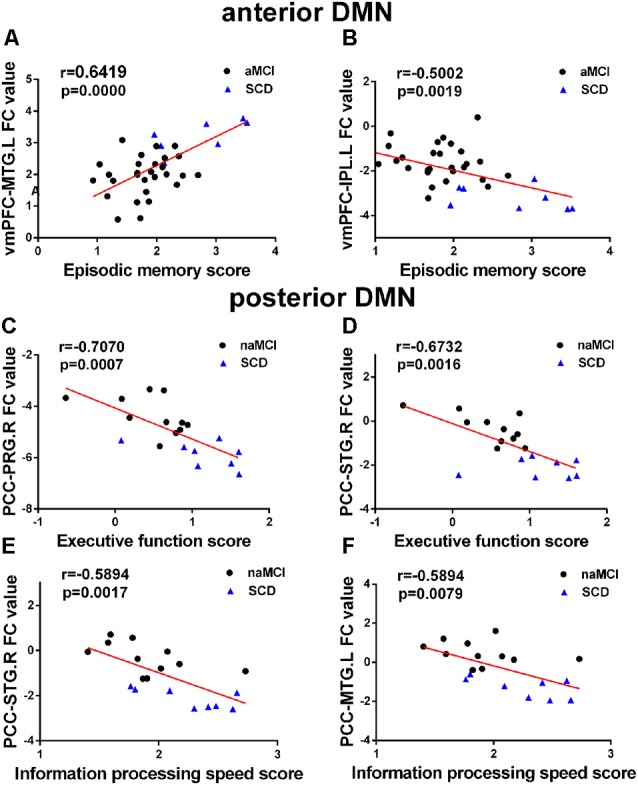
**(A–F)** Significant associations between altered FC and cognitive function including episodic memory, executive function, and information processing speed in anterior DMN (aDMN) and posterior DMN (pDMN) (Bonferroni corrected, *p* < 0.05). Age, gender, and years of education were used as covariates for all these results. aMCI, amnestic mild cognitive impairment; naMCI, non-amnestic mild cognitive impairment; SCD, subjective cognitive decline; vmPFC, ventral medial prefrontal cortex; PCC, posterior cingulate cortex; MTG, middle temporal gyrus; PRG, precentral gyrus; STG, superior temporal gyrus; IPL, inferior parietal lobule; L, left; R, right.

A correlation analysis was conducted between regions with altered FC and cognitive domains (Bonferroni corrected, *p* < 0.05). In the groups consisting of SCD and aMCI, the analysis showed that the altered FC between the vmPFC and the left IPL in the aDMN is negatively correlated with episodic memory (*r* = −0.5002, *p* = 0.0019), while the altered FC between the vmPFC and the left MTG is positively correlated with episodic memory (*r* = 0.6419, *p* = < 0.0001). In the groups that contained SCD and naMCI, the analysis showed significant negative correlation in the pDMN. Altered FC between the PCC and the right STG was negatively correlated to both executive function (*r* = −0.6732, *r* = 0.0016) and information processing speed (*r* = −0.5894, *p* = 0.0017). Altered FC between the PCC and the right PRG was negatively correlated with executive function (*r* = −0.7070, *p* = 0.0007), and altered FC between the PCC and the left MTG was negatively correlated with information processing speed (*r* = −0.5894, *p* = 0.0079). Age, gender, and years of education were used as covariates for all these results. There was no statistically significant correlation (Bonferroni corrected, *p* < 0.05) between the cognition domains and the remaining areas (see Figure 3).

## Discussion

The present study aimed to investigate changes in FC of the anterior and posterior DMN between different groups (HC, SCD, naMCI, and aMCI) and to explore how this altered FC influences cognitive function. Corresponding with our hypothesis, our study presents two main findings. First, the FC of the anterior and posterior subnetworks in the DMN was damaged in the SCD, naMCI, and aMCI groups after controlling the effects of age, gender, level of education, and GM volumes. Second, the correlation analyses demonstrated that the altered connectivity patterns of the DMN subnetworks were associated with impaired cognitive function. Moreover, our study further confirms that the aDMN and pDMN are functionally independent, and the altered subnetworks have different effects on cognitive function.

The current study indicates that there is significantly altered FC in the SCD, naMCI, and aMCI groups in/between the anterior DMN and the posterior DMN, thereby proving the heterogeneity of the DMN (Damoiseaux et al., 2012; Yuan et al., 2016b). Compared to the HC, the naMCI and aMCI groups showed increased FC both in the aDMN and pDMN, while the SCD group showed increased FC in the aDMN and decreased FC in the pDMN. Over the years, alterations in FC in the DMN have been a focal point of many studies, but few investigations have examined changes in FC in the specific DMN subnetworks.

Our findings in the three preclinical stages of AD demonstrate a widespread alteration of FC in the DMN, and further illustrate that AD is a disconnection syndrome (Palesi et al., 2016). Specifically, our results indicate that patients with aMCI have significantly increased FC between the vmPFC and bilateral IPL (Cai et al., 2017), while naMCI patients have significantly increased FC between the vmPFC and left IPL compared with the HC. The IPL is a heterogeneous brain area with a role in multiple-modality functions including sensory motor processing, salience detection, executive control, and especially episodic memory (Wang et al., 2012, 2016, 2017; Yuan et al., 2016a). Therefore, our findings might indicate that the right IPL is one of the brain areas that is responsible for episodic memory. It is worth noting that the SCD group showed increased FC between the vmPFC and the bilateral SFG in the aDMN, while it showed decreased FC between the PCC and the right precuneus in the pDMN compared to the HC, proving that the changes in FC precede the appearance of clinical manifestations (Hayes et al., 2017). Notably, the alterations in the precuneus may begin as early as approximately 10–20 years before the onset of clinical symptoms of dementia (Bateman et al., 2012). Furthermore, the only significantly decreased FC revealed by our study is the FC between the PCC and the right precuneus in the SCD group compared with the HC, suggesting that the right precuneus might be the first damaged area in the DMN and that the increased FC between the vmPFC and the bilateral SFG might be a compensatory response. However, no altered FC was found between the PCC and the precuneus in the naMCI and aMCI groups compared to the HC, which might represent a characteristic change in the SCD patients. The PCC-precuneus plays a vital role in the DMN, and they are among the brain regions most prone to AD because of their connective, metabolic, and vascular characteristics (Cha et al., 2013; Prieto Del Val et al., 2016; Wu et al., 2016; Wang et al., 2019). In addition, the present study shows that the naMCI group exhibited increased FC between the PCC and the left MTG, while the aMCI group showed increased FC between the PCC and the right MFG compared to the HC (Liang et al., 2011; Cha et al., 2013). The MFG region consists of a caudal and rostral area, the latter including a part of the dorsal lateral prefrontal cortex, which is responsible for working memory and executive cognitive functions (Barbey et al., 2013). A previously published study reported that the MFG is an important area and that increased FC in the right MFG might be a compensation mechanism (Cha et al., 2013). Interestingly, the aMCI group showed decreased FC between the PCC and the right CPL compared to the SCD group, while both the SCD and aMCI groups showed no significant differences when compared to the HC. The cerebellum is involved not only in movement and balance but also in advanced cognitive functions (Gottwald et al., 2003). The decreased FC between the PCC and the cerebellum suggests a potential effect on the cerebellar-related cognitive functions in aMCI; this finding is in accordance with our previous studies (Chen et al., 2015, 2019b; Yang et al., 2017). Taken together, the increased FC between the vmPFC and the left IPL and between the PCC and the right MFG and the left MTG could serve as a biomarker for identifying patients with naMCI and aMCI. In addition, the altered FC between the PCC and the precuneus could be a potential biomarker of SCD.

The present study indicates that the SCD group showed increased FC in the aDMN and decreased FC in the pDMN, while the naMCI and aMCI groups showed increased FC both in the aDMN and the pDMN. We speculate that memory damage in the SCD patients is not obvious and that there may be a decrease in FC in the pDMN. With the progressive decline of cognitive ability, FC in the naMCI and aMCI patients gradually increases, which may be indicative of compensatory activity (Qi et al., 2010). We can conclude that SCD is the intermediate stage between healthy elderly and MCI, and both naMCI and aMCI are the preclinical stages of AD. Additionally, the results of the present study show that the DMN is an important intrinsic network to differentiate between the healthy elderly, SCD, naMCI, and aMCI (Wang et al., 2013).

The present study showed observably positive and negative correlations between FC and cognitive domain scores in patients with SCD, naMCI, and aMCI. In the anterior DMN subnetwork, a noteworthy finding was that the episodic memory score negatively correlated with the increased FC between the vmPFC and the left IPL in the SCD and aMCI patients; this finding confirms previously published articles stating that altered FC is associated with memory deficits in patients with aMCI (Bai et al., 2009; Ofer et al., 2018). Furthermore, the finding indicates that the increased FC between the vmPFC and the left IPL may be an attempt to combat the induced functional decline and the negative association with episodic memory, and this may be reflective of compensatory attempts (Bai et al., 2009; Zhang et al., 2016). It is worth noting that the increased FC in the aDMN between the vmPFC and the left MTG in SCD patients is positively correlated with episodic memory, suggesting that episodic memory in SCD patients is not impaired. This could be important evidence that the SCD is an intermediate stage between healthy elderly and MCI. In the posterior DMN, we found that the FC in the left MTG, the right PRG, and the right STG is significantly negatively correlated with cognitive function, including the information processing speed and the executive function score. It has been reported that the left MTG is significantly related to MMSE scores, and this could reflect the progression of AD (Cha et al., 2013).

In terms of subnetworks, our findings show that episodic memory is significantly correlated with the altered aDMN, while information processing speed and executive function are negatively correlated with the pDMN. These results indicate that the aDMN mainly controls the episodic memory function, while the pDMN is primarily responsible for other cognitive functions except for memory.

## Limitations

There are two major limitations in our present study. First, significant differences in age and education levels were present among the four groups and this potentially negatively affected our results. However, to avoid the effects of these confounding factors, we performed all statistical analyses with age, education level, and gender as covariates. Therefore, we believe that our results are credible.

Second, the study had a cross-sectional design and a small sample size, which may prevent us from detecting smaller effect sizes and may lead to some null results. We will continue to recruit volunteers to participate in this study and perform regular follow up in the future. We will try to avoid all possibilities of potential bias in our data and to further confirm our results.

## Conclusion

This study demonstrates that SCD, naMCI, and aMCI are three preclinical stages of AD and that they exhibit distinct alternations in the FC of DMN subnetworks. Furthermore, the patient group data showed that the altered FC affects cognition, including episodic memory, executive function, and information processing speed. These results can provide novel insights for tailored clinical intervention across the preclinical AD spectrum.

## Data Availability Statement

The datasets analyzed in this manuscript are not publicly available. Requests to access the datasets should be directed to ericcst@aliyun.com.

## Ethics Statement

The studies involving human participants were reviewed and approved by the responsible Human Participants Ethics Committee of the Affiliated Brain Hospital of Nanjing Medical University. The patients/participants provided their written informed consent to participate in this study. Written informed consent was obtained from the individual(s) for the publication of any potentially identifiable images or data included in this article.

## Author Contributions

CXu, BY, CXi and JC: designed the study. CXu, BY, CXi, JC, YY, JX, SW, MW, NJ, XZ, YZ, JR and WY: collected the data. CXu and BY: analyzed the data and prepared the manuscript.

## Conflict of Interest

The authors declare that the research was conducted in the absence of any commercial or financial relationships that could be construed as a potential conflict of interest.

## Footnotes

^1^http://www.mathworks.com/products/matlab/

^2^https://www.fil.ion.ucl.ac.uk/spm/

^3^http://resting-fmri.sourceforge.net
